# B-cell epitope prediction through a graph model

**DOI:** 10.1186/1471-2105-13-S17-S20

**Published:** 2012-12-07

**Authors:** Liang Zhao, Limsoon Wong, Lanyuan Lu, Steven CH Hoi, Jinyan Li

**Affiliations:** 1 Bioinformatics Research Center, School of Computer Engineering, Nanyang Technological University, Singapore; 2 School of Computing, National University of Singapore, Singapore; 3 School of Biological Science, Nanyang Technological University, Singapore; 4 Advanced Analytics Institute, School of Software, Faculty of Engineering and IT, University of Technology Sydney, PO Box 123, NSW 2007, Australia

## Abstract

**Background:**

Prediction of B-cell epitopes from antigens is useful to understand the immune basis of antibody-antigen recognition, and is helpful in vaccine design and drug development. Tremendous efforts have been devoted to this long-studied problem, however, existing methods have at least two common limitations. One is that they only favor prediction of those epitopes with protrusive conformations, but show poor performance in dealing with planar epitopes. The other limit is that they predict all of the antigenic residues of an antigen as belonging to one single epitope even when multiple non-overlapping epitopes of an antigen exist.

**Results:**

In this paper, we propose to divide an antigen surface graph into subgraphs by using a Markov Clustering algorithm, and then we construct a classifier to distinguish these subgraphs as epitope or non-epitope subgraphs. This classifier is then taken to predict epitopes for a test antigen. On a big data set comprising 92 antigen-antibody PDB complexes, our method significantly outperforms the state-of-the-art epitope prediction methods, achieving 24.7% higher averaged f-score than the best existing models. In particular, our method can successfully identify those epitopes with a non-planarity which is too small to be addressed by the other models. Our method can also detect multiple epitopes whenever they exist.

**Conclusions:**

Various protrusive and planar patches at the surface of antigens can be distinguishable by using graphical models combined with unsupervised clustering and supervised learning ideas. The difficult problem of identifying multiple epitopes from an antigen can be made easied by using our subgraph approach. The outstanding residue combinations found in the supervised learning will be useful for us to form new hypothesis in future studies.

## Background

A B-cell epitope is a set of spatially proximate residues in an antigen that can be recognized by antibodies to activate immune response [1]. B-cell epitopes are of two types: about 10% of them are linear B-cell epitopes and about 90% are conformational B-cell epitopes [2-4]. Linear epitopes differ from conformational epitopes in the continuity of their residues in primary sequence--residues of a linear-epitope are contiguous in primary sequence while the residues in a conformational-epitope are not. B-cell epitope prediction is a long-studied problem of high complexity which aims to identify those residues in an antigen forming one or multiple epitopes.

This problem has attracted tremendous efforts over the last two decades because of its significance in prophylactic and therapeutic biomedical applications [5]. Various approaches have been proposed to identify conformational epitopes, for example, by clustering accessible surface area (ASA) [6], by combining residues' ASA and their spatial contact [7], by grouping surface residues under their protrusion index [8], by aggregating epitope-favorable triangular patches [9], or by using naïve Bayesian classifier on residues' physicochemical and geometrical properties [10]. Far more approaches have been developed for predicting linear epitopes. Some of these methods use just a single feature of residues--such as hydrophobicity, polarity, or flexibility only--to detect the crests or troughs of propensity values as epitopes [11,12]. The other methods take complicated machine learning approaches, including artificial neural network, Bayesian network, and kernel methods, to tackle this problem [13-19]. With these tremendous efforts, this field of research has been advanced significantly and the best AUC performance has reached to 0.644 [9]. However, there are still many limitations in existing methods, and huge room for performance improvement exists.

**Figure 1:**
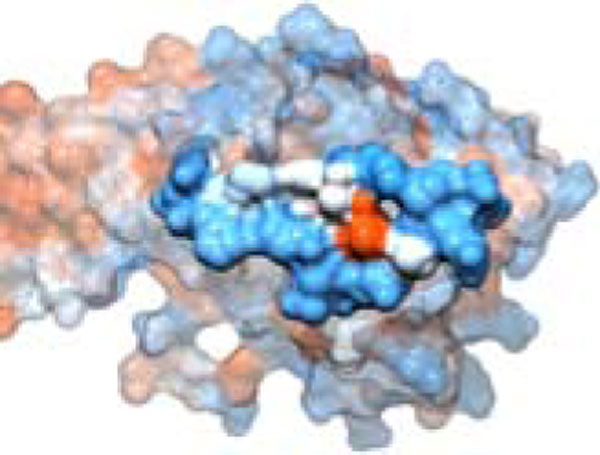
**A hydrophilic island with a hydrophobic core in the binding site of paracoccus denitrificans cytochrome C oxidase**. Interacting with an antibody (PDB complex 1AR1). The hydrophilic residues are rendered by blue and the hydrophobic residues are colored by orange. The shade of colors represents hydrophobic intensity. This figure is produced by using Chimera [33].

**Figure 2:**
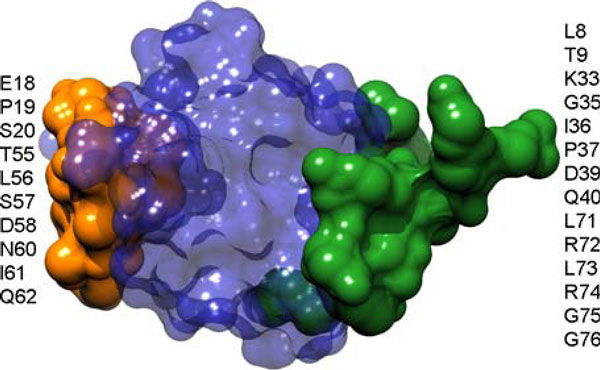
**An example of a multiple-epitope antigen**. The ten residues with color orange are the residues of the epitope on the ubiquitin antigen (chain X) in PDB complex 3DVG, while the fourteen residues colored in forest green are the residues of the epitope on the ubiquitin antigen (chain Y) in PDB complex 3DVG.

A limitation of those methods using geometrical properties [7,8,10] is that they only favor epitopes with protrusive shapes, not identifying epitopes in other formations such as planar shapes. In fact, many epitopes are shaped at plain areas of antigens. For example, the surface atoms of the epitope of paracoccus denitrificans cytochrome C oxidase is very at in 3-dimensional space with a root mean square deviation (rmsd, an index of non-planarity) of only 1.08Å (Figure 1). The second limitation of the conventional methods is that they do not separate or distinguish between any two epitopes in an antigen when multiple epitopes exist. They only tell which residue of the antigen is antigenic, but not tell to which epitope it belongs to. That is, only a union of all antigenic residues, irrespective to specific epitopes, are just predicted. This is a limitation because multiple epitopes are possible at the same antigen [20]. For instance, there exist two non-overlapping epitopes on the ubiquitin antigen: one of them has a very smooth surface with a non-planarity of 1.04Å, while the other stretches out remarkably with a non-planarity of 3.14Å. See Figure 2 for more details of their constituent resides. In this work, we propose a graph-based model to improve the prediction performance by identifying both protrusive and planar epitopes and by detecting multiple epitopes in an antigen if applicable (i.e., identifying all of the epitopes instead of the union of all epitope residues).

The use of graph model is motivated by the following biological observations. First, the tight packing of residues at each protein surface can be effectively represented by a graph. Second, epitope/non-epitope residues form particular patches separately on antigen surfaces, displaying distinct subgraphs of their own characteristics. As shown in Figure 1, the binding site shapes like a hydrophilic island (a hydrophilic subgraph) containing a hydrophobic core (a hydrophobic subgraph). It can be also seen that this island subgraph is surrounded by hydrophobic non-epitope residues which form a non-epitope subgraph. Third, the distinction between protrusive and planar eptiopes can be manifested by the change of weights in the connections between residues.

Our graph-based prediction method consists of three main steps: construct a weighted graph to represent an antigen surface, cluster the nodes of this weighted graph, and learn a label (epitope or non-epitope) for each cluster. Specifically, we take the idea of Delaunay tessellation and use Qhull [21] in the implementation of Delaunay tessellation to construct a protein surface graph. The weights of the edges in this graph are determined by $X^{2}$ test statistics combined with a log odds ratio of each edge type. An edge type is determined by the amino acid types of the interacting residue pair. Then, a Markov CLustering algorithm (MCL) [22] is used to partition the entire graph into subgraphs based on the weights of the edges and the graph topology. MCL simulates flows in a network with two operations: expansion and inflation. Expansion increases homogeneity of nodes within one subgraph, while inflation evaporates inter-flow between different subgraphs and amplifies flow within subgraphs. These ideas mimic properties of residues connecting within an epitope, within a non-epitope, or between an epitope and a non-epitope. Thus, we can divide the weighted antigen surface graph into a good set of subgraphs for the subsequent learning algorithms to predict these subgraphs as epitopes or non-epitopes accurately.

Experimental results on a set of 92 non-redundant antibody-antigen complexes compiled from the Protein Data Bank (PDB) [23] show that our proposed graph model improves the performance of B-cell epitope prediction significantly and, it is also able to identify multiple epitopes as well as predict epitopes with various geometrical formations. For ease of reference, we refer to the proposed **B**-Cell **e**pi**Top**e prediction model as **BeTop**. Our data and web server for B-cell epitope prediction are available at http://sunim1.sce.ntu.edu.sg/~s080011/betop/index.php.

## Materials and methods

### Collection of antigen protein data

Protein complexes satisfying the following criteria were retrieved from the PDB on May 14th, 2011: (i) the macromolecular type is protein only, no DNA, RNA, or their hybrid complexes; (ii) the number of protein chains in an asymmetric unit of one complex is larger than two; (iii) the length of every chain is larger than or equal to 30; (iv) the X-ray resolution of one complex is less than 3Å; and (v) the structure title contains at least one of the following terms: antibody, Fab, Fv, or VHH. We obtained 622 antibody-antigen complexes. As transformed and redundant chains in the raw PDB complexes may cause noise effect on the results, we removed all of the transformed chains and duplicate chains. One antigen chain is considered as a duplicate if there exists one pair-wise chain similarity between this chain and one of the other in the data set larger than 80%, a threshold widely used to remove redundant antigens [24]. Removal of duplicate chains by pair-wise chain similarity may filter out multi-epitope antigens, but it can significantly reduce more noise data because the number of non-epitope residues is extremely larger than the number of epitope residue for an antigen. Asymmetric units in each complex that do not have structural difference were also excluded from our consideration. Finally, a non-redundant data set containing 92 antibody-antigen complexes were collected for our model training and testing. Epitope residues on antigen surfaces were determined by using the Euclidian distance of 4Å for every antigen-antibody PDB complex, following the traditional method for determining epitope residues [7].

### Construction of epitope prediction model

The training phase of our prediction method has the following steps: (i) antigen surface triangulation, (ii) weight calculation for edges, (iii) clustering on the nodes of the graphs, and (iv) supervised learning for distinguishing between epitope subgraphs and non-epitope subgraphs. The details of each step are presented below.

#### Triangulation of an antigen surface

**Figure 3:**
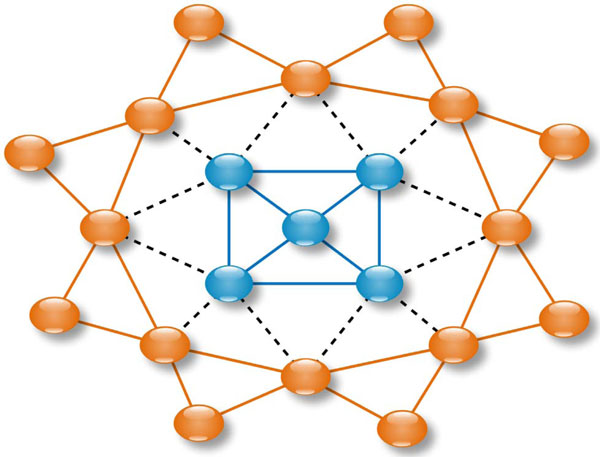
**Diagram of an antigen surface graph**. Nodes are residues and edges represent residues' spatial relation. Blue nodes are epitope residues, and orange ones are non-epitope residues. Dash lines are boundary edges between two clusters, while solid lines are edges within a cluster.

A surface graph of an antigen structure is built in two steps: determine the surface atoms of the antigen, and then build an atom-level graph for these surface atoms and upgrade into a residue-level graph. To obtain surface atoms of an antigen with 3D coordinates, we compute each atom's ASA by using NACCESS [25] with the default probe size. Those atoms with ASA ≥ 10*Å*^2 ^are defined as surface atoms. A graph of these surface atoms is constructed as per Delaunay triangulation rule which has been commonly used to construct protein surface graph [26]. To upgrade an atom-level graph into a residue-level graph, we ignore connections of the atoms within the same residue, e.g., ignore connection between *C_α _*and *C_β _*of Alanine; and then merge multiple atom connections between two different residues into one edge, e.g. merge the connection between *O*_*D*1 _of Aspartate and *C*_*G*1 _of Isoleucine and the connection between *O*_*D*2 _of Aspartate and *C*_*G*2 _of Isoleucine into one edge. Atom connections that have Euclidian distances larger than 6Å are also removed. Then, residues are distinguished by their positions--i.e., two residues are considered different if they have different positions even when they are of the same amino acid type. Figure 3 shows a graph of an antigen after triangulation, in which nodes are surface residues and edges represent residues' spatial relations.

#### Weight calculation for edges

The weight between two residue types *x *and *y *within an epitope subgraph or within a non-epitope subgraph in our graph database is given by

(1) $$W_{xy}=\alpha \cdot {\bar{W}}_{xy}^{\chi^{2}}+\left(1-\alpha \right)\cdot {\bar{W}}_{xy}^{L},$$

where $\bar{W}$ is the normalized value of *W*, and $W_{xy}^{X^{2}}$ and $W_{xy}^{L}$ are the $X^{2}$ test and the log odds ratio of the frequencies of *xy *(edge between *x *and *y*) between epitope clusters and non-epitope clusters, respectively. $W_{xy}^{X^{2}}$ and $W_{xy}^{L}$ are calculated by using

(1a) $$W_{xy}^{\chi^{2}}=\underset{c}{\sum }{\left(N_{xy}^{c}-E_{xy}^{c}\right)}^{2}/E_{xy}^{c}$$

(1b) $$W_{xy}^{L}=\text{log}\;\left(P_{xy}/Q_{xy}\right)$$

where *c *∈ {*epitope, non*-*epitope*}, $N_{xy}^{c}$ is the number of edges with type *xy *and label *c *in our training data, $E_{xy}^{c}$ is the number of expected edges with type *xy *and label *c, P_xy _*is the probability that a pair of residues *xy *in epitopes, and *Q_xy _*is the probability that a pair of residues *xy *in non-epitopes. *P_xy _*is given by

$$P_{xy}=N_{xy}/\underset{x^{\prime }}{\sum }\underset{y^{\prime }}{\sum }N_{x^{\prime }\;y^{\prime }},$$

where, *N_xy _*is the number of residue pairs *xy *in a cluster, i.e., the number of edges connecting two nodes with one node labeled as *x *and the other as *y*. *Q_xy _*is calculated by the same way of computing *P_xy_*.

The weight calculation for boundary edges is very innovative. A boundary edge is an edge connecting an epitope residue and a non-epitope residue. We group all of the boundary edges (e.g. dashed black lines in Figure 3) in our graph database as a class, and take all epitope edges (e.g. solid blue lines in Figure 3) as the other class. Then, we apply Equation (1a) and (1b) to calculate the weights $W_{xy}^{\prime }$ for the boundary edges by setting *c *∈ {*boundary_class, epitope_classg*}. This process is also applied with regard to the boundary class and non-epitope class (e.g., edges with solid orange lines in Figure 3) to determine weights $W_{xy}^{\prime \prime }$ for the boundary edges. In other words, $W_{xy}^{\prime }$ and $W_{xy}^{\prime \prime }$ are determined by using the exactly same equations as computing *W_xy_*, with substitution of the relevant class label *c*. The weight of a boundary edge *xy *is finally set as $W_{xy}^{\prime }$ or $W_{xy}^{\prime \prime }$ whichever is larger. Those boundary edges with heavy weights (larger than a threshold *W*_0_) are *definitely *boundary edges between epitope and non-epitope subgraphs. We remove them to sharpen the distinction in the later clustering step and supervised learning. Boundary edges might change to another set when different computational methods are used to define epitope residues, such as using accessible surface area larger than 1Å^2 ^upon binding with an antibody [6,27] and distance threshold of 4Å [7,28], 5Å [29] or 6Å [30]. However, Ponomarenko *et. al*. have shown that epitope residues have no significant difference when various criteria are used to capture epitope residues [24].

As a few number of large weights can pull all weight values towards zero after normalization, we further contrast normalized weights *W_xy _*to amplify important weights and suppress trifling weights by

$$f\left(W\right)=\frac{1}{1+{\left(\theta \frac{W}{1-W}\right)}^{-\gamma }}.$$

where *θ *and are *γ *optimized as 3 and 3 in this study.

Since there are only 20 different standard residue types, the total number of different weights between two residue types is $210\;\;\left(=\;{\text{C}}_{2}^{20}+{\text{C}}_{1}^{20}\right)$.

#### Clustering on nodes in an antigen surface graph

Antigen surface graphs are constructed by Qhull with weights *W *on edges determined by the procedure above. We then use mcl [22] (an implementation of the MCL algorithm with inflation coefficient *r *of 1.8) to cluster the nodes and edges of every antigen graph into subgraphs. In the MCL algorithm, the graph of an antigen surface residues is represented by a square matrix *M*, where each row/column represents a surface residue and the value of each entry is the weight of these two residue types. In the expansion stage of MCL, *M *is expanded as the normal product of itself; during the inflation stage, the matrix *M *undertakes Hadamard power with coefficient *r *followed by normalization. This two steps keep on in iteration until an equilibrium state is reached, i.e., when expansion and inflation do not alter the matrix any more.

The subgraphs of an antigen surface clustered by MCL are not always clean and some subgraphs may contain a mixture of epitope residues and non-epitope residues. To clean up the training data, we consider a subgraph as an epitope subgraph if the number of epitope residues in this subgraph is larger than the number of non-epitope residues and, as a non-epitope subgraph if no or very few (say, at most two) epitope residues show up. Subgraphs with other situations are considered as noise data which are overlooked during model training. We adopt this strategy because of the small number of epitope residues. We note that this approach is tolerant to false positives, but is sensitive to false negative.

#### Supervised learning for distinguishing epitope subgraphs and non-epitope subgraphs

By using mcl, each antigen surface graph is clustered into a number of subgraphs. To distinguish between epitope subgraphs and non-epitope subgraphs, we design a feature vector to represent all of these subgraphs in our training data. Each subgraph is transformed into a feature vector with 1770 dimensions, which comprises $20\;\left(=\;C_{1}^{20}\right)$ dimensions of single residues, $210\;\;\left(=C_{1}^{20}+C_{2}^{20}\right)$ dimensions of residue pairs, and $1540\;\left(=C_{1}^{20}+C_{2}^{20}\cdot C_{1}^{2}+C_{3}^{20}\right)$ dimensions of residue triangles. A single-residue feature takes the weighted summation of $X^{2}$ test and log odds ratio on the frequencies of the residue type between epitope clusters and non-epitope clusters, which is similar to the calculation of the weight of a pair of residue types shown in Equation (1). A residue-pair feature takes the weight of this edge in the subgraph as its value, and a triplet feature takes the average weight of the three edges in the subgraph as its value.

The number of nodes in a subgraph is very small (15 on average); but the dimension of each vector is very large (1770). Therefore, each vector is very sparse and, some features even have no differentiability between epitope subgraphs and non-epitope subgraphs. Hence, feature selection is conducted to maximize classification performance. The feature selection was done by using the LIBSVM [31] feature-selection module targeting at maximizing classification f-score. As a result, 144 from the 1770 features are chosen for classifying epitope subgraphs from non-epitopes subgraphs.

Due to the extreme imbalance between the epitope residue number and non-epitope residue number for an antigen surface graph (15 and 120 on average in our data set), the number of non-epitope subgraphs far exceeds the number of epitope subgraphs as generated by mcl. To address this imbalance problem, a two-stage supervised learning, multi-SVM classification and trust-reliable voting, is taken to accomplish the distinction between epitope and non-epitope subgraphs. The number of SVM classifiers is automatically determined by the proportion between non-epitope subgraphs and epitope subgraphs. Based on our data set in this work, the number of SVM classifiers is nine. For each classifier in the first stage, a parameter grid-search is carried out on a balanced training data set to maximize model performance, while in the second stage the final decision is voted and determined by

(2) $$y=sgn\left(\underset{i}{\sum }w_{i}\cdot f\left(x_{i}\right)\;\cdot \;\delta_{i}-\theta_{0}\right),$$

where

$$\begin{array}{c} \delta_{i}=1-g\left(\tau_{0}-|p_{x_{i}}^{0}-0.5|\right)\;\cdot \;h\left(\underset{i}{\sum }sgn\left(p_{x_{i}}^{0}-0.5\right)\right)\\ g\left(x\right)=\left\{\begin{array}{c} 1,\;x>0\\ 0,\;x\leq 0 \end{array}\right)\;\;\;\;\;h\left(x\right)=\left\{\begin{array}{c} 1,\;x\neq 0\\ 0,\;x=0 \end{array}\right)\;\;\;\;\;sgn\left(x\right)=\left\{\begin{array}{c} \;\;\;1,\;\;x\;\;>\;\;0\\ -1,\;\;x\;\;<\;\;0 \end{array}\right), \end{array}$$

and the symbol annotations are as follows:

• *y*: epitope/non-epitope label for a sample predicted by the model;

• *w_i_*: weight of classifier *i *computed by its performance;

• *f*(*x_i_*): label for a sample *x *determined by classifier *i *in the first level;

• $p_{x_{i}}^{0}$: probability of classifier *i *that predicts sample *x *as non-epitope;

• *δ_i_*: determinant of classifier *i*. *δ_i _*is 0 when the classifier *i *is dubious and other confident classifiers exist.

*θ*_0 _is a threshold to filter out non-epitope residues, and *τ*_0 _is used to control to what extent we trust the classifier.

### Prediction of epitopes for an unknown antigen

Given an antigen with 3D coordinate information, the following steps are taken to identify one or multiple epitope for this antigen: (i) calculate each atom's ASA by using NACCESS, and filter out those atoms with ASA less than 10Å^2^; (ii) construct an atom-level graph by using Qhull and upgrade it to a residue-level graph; (iii) assign weights to all edges of this residue graph, where the weights are those determined during the training; (iv) cluster this undirected and weighted graph into exclusive subgraphs using mcl; and (v) transform every subgraph into a feature vector, and predict its label by the well-trained two-stage classification model. Epitope residues are the residues within those subgraphs which are predicted as epitope. Two epitope subgraphs can be merged together if they are connected in the original surface graph.

## Results and discussions

Our graph-based method BeTop made remarkable improvement on B-cell epitope prediction in comparison to the state-of-the-art methods. First, BeTop shows significant improvement on overall prediction accuracy. Second, BeTop is capable of predicting epitopes located at both protrusive and planar surface areas. Third, BeTop is able to identify multiple epitopes if an antigen contains them. The detailed results of all these are presented below together with highlights of those features that distinguish epitope subgraphs from non-epitope subgraphs.

### Significant improvement of prediction accuracy

Four performance metrics are adopted to evaluate model performance--viz., sensitivity (sen), specificity (spe), f-score, and accuracy (acc). They are defined as *sen *= *TP/*(*TP *+ *FN*), *spe *= *TN/*(*TN *+ *FP*), *f*-*score *= 2*pre*sen/(*pre *+ *sen*), and *acc *= (*TP *+ *TN*)/(*TP *+ *FP *+ *TN *+ *FN*), where *TP, TN, FP*, and *FN *represent the number of predicted true positive, true negative, false positive and false negative samples, respectively. Due to the imbalance nature in the composition of non-epitope residues and epitope residues in an antigen, accuracy is not competent to measure model performance. Instead, f-score is more appropriate to evaluate BeTop's performance and to compare with other models.

**Figure 4:**
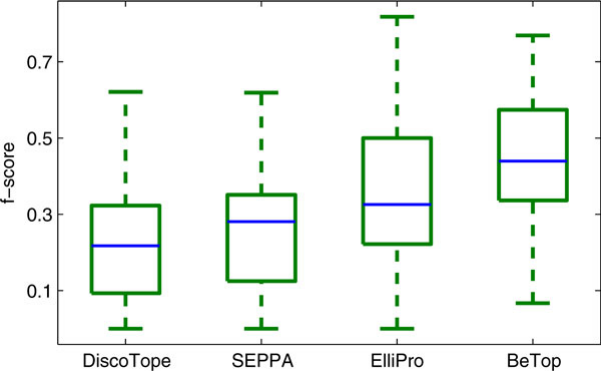
**B-cell epitope prediction performance by BeTop, DiscoTope, SEPPA, and ElliPro**. The optimal parameters *α, θ*_0 _and *τ*_0 _are set to 0.3, 0.3 and 0.05, respectively.

Ten fold cross validation is applied to measure the overall performance of BeTop on the 92 non-redundant antigen-antibody PDB complexes. The f-score comparison between BeTop and the state-of-the-art epitope prediction methods DiscoTope [7], SEPPA [9] and ElliPro [8] are shown in Figure 4. We note that ElliPro can produce a short list of candidate epitopes. Its performance reported here is summarized based on its best result among all of the predicted candidates for each antigen. In the case that BeTop identifies multiple epitopes for an antigen, its performance is reported in the same way as ElliPro for a fair comparison. From Figure 4, it can be seen that BeTop outperforms all existing models significantly. The f-score *t*-test *p*-values between BeTop and the other models are shown in Table 1 to illustrate the significance level that BeTop is better.

**Table 1:** F-score *t*-test *p*-values between BeTop, DiscoTope, SEPPA and ElliPro.

	DiscoTope (0.22 *± *0.14)	SEPPA (0.25 *± *0.16)	ElliPro (0.36 *± *0.20)	BeTop (0.45 *± *0.16)
DiscoTope		1.6e-1	7.9e-8	1.8e-17
SEPPA			2.3e-5	7.3e-15
ElliPro				2.0e-3

The averaged sensitivity, specificity, accuracy and AUC values for DiscoTope, SEPPA, ElliPro and BeTop are shown in Table 2. It is clear that BeTop is remarkably better than other models in terms of sensitivity, accuracy and AUC. The specificity of BeTop is slightly lower than that of ElliPro, but this value is much better than the other two models. More detailed results for each antigen in terms of sensitivity, specificity, f-score and accuracy can be found in the supplementary material Additional File 1: Table S1.

**Table 2:** The averaged performances comparison between BeTop, DiscoTope, SEPPA and ElliPro on sensitivity, specificity, accuracy and AUC.

	DiscoTope	SEPPA	ElliPro	BeTop
sensitivity	0.377 *± *0.278	0.526 *± *0.345	0.501 *± *0.290	**0.665 ***± ***0.239**
specificity	0.686 *± *0.168	0.665 *± *0.255	**0.849 ***± ***0.137**	0.809 *± *0.162
accuracy	0.631 *± *0.133	0.659 *± *0.193	0.798 *± *0.126	**0.802 ***± ***0.134**
AUC	0.531 *± *0.127	0.595 *± *0.157	0.675 *± *0.140	**0.737 ***± ***0.107**

One of the novel ideas used in this study is reducing the weight of boundary edges to distinguish epitope from non-epitope. Thus, we further compare the performances of BeTop with suppressing weights of boundary edges and without suppressing weights of boundary edges. Experimental results show that the averaged f-scores are 0.45 and 0.41 for the two situations, with increment of f-score by 8.9%. The *t*-test *p*-value of 0.11 between the two sets of f-scores also demonstrates the improvement of performance by decreasing weights of edges enriched in boundary class.

### Locating epitopes with planar formations

Existing conformational epitope prediction methods such as [7,8,10] heavily rely on the spatial structure information and non-planarity properties of antigens. They usually have a good performance on epitopes that have a protrusive surface, otherwise the performance becomes poor. To understand the effect of non-planarity of epitopes on epitope prediction, we divide all of the epitopes in our database into groups based on a non-planarity index. The non-planarity of a residue cluster is measured by the root-mean-square deviation of all the surface atoms of this cluster of residues. It is expected that those structure-based prediction models favor epitopes with large non-planarity but not at epitopes.

**Figure 5:**
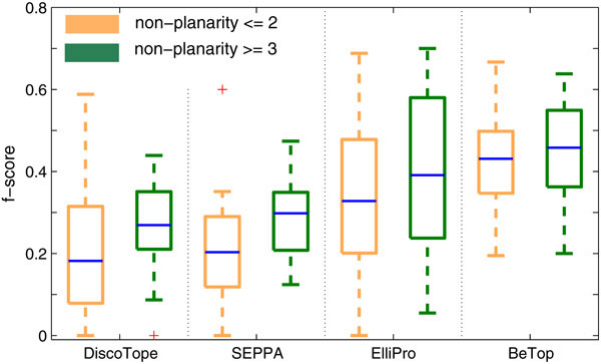
**Performance comparison at different levels of epitope non-planarity**.

Our experimental result is shown in Figure 5. It is clear that BeTop works very well with an average f-score 0.432 on at epitopes, namely on those epitopes having a non-planarity less than 2Å. However, DiscoTope, SEPPA and ElliPro all had difficulties to detect such epitopes with f-scores of only 0.214, 0.207, and 0.337 respectively.

Taking PDB entry 1AR1 as example again (Figure 1), its epitope consists of 19 residues, and the non-planarity of this epitope is as small as 1.08Å, indicating a very flat surface area. The f-score achieved by BeTop is 0.88 (with 16 true positives and 1 false positive). However, ElliPro, SEPPA, DiscoTope made an f-score of 0.273 (with 7 true positives and 22 false positives), 0.000, and 0.000, respectively. As another example, the prediction performance by BeTop, ElliPro, SEPPA and DiscoTope on the epitope residues of PDB entry 1N8Z are 0.667, 0.194, 0.198 and 0.07, respectively. This epitope also has a very planar surface with non-planarity of 1.88Å.

For epitopes having a large non-planarity bigger than or equal to 3Å, BeTop also performs better than the other models. The f-score is improved by 65.6%, 55.7% and 11.8% over DiscoTope, SEPPA and ElliPro, respectively. In particular, in comparison to ElliPro, which detects twisted epitopes based on residues' protrusion index, BeTop still achieved a better performance.

In summary, the f-score of the 3 existing methods becomes poor when the non-planarity of epitopes becomes flat. However, BeTop performs equally well under both protrusive and planar conditions, demonstrating that our proposed BeTop graph model is invariant to the change of epitope non-planarity.

### Identifying multiple epitopes from an antigen

Although BeTop is trained on single-epitope antigen-antibody complexes, the framework has no limitation on the number of predicted epitopes. To evaluate BeTop's capability in identifying multiple epitopes in an antigen, we tested it on a data set of epitopes that are comprehensively explored in [20].

The multiple epitopes of these antigens are determined by the following steps: (i) determine epitope residues for each complex by using the 4Å Euclidian distance criteria between the antigen and antibody; (ii) calculate a pair-wise epitope similarity between two complexes *X *and *Y *of the same antigen by using *S_XY _*= |*X *∩ *Y *|/*min*(|*X*|, *|Y|*); (iii) cluster epitopes based on their similarities for each antigen; (iv) select representative epitopes for each cluster with the best resolution, and map all representative epitopes to one of them with the finest resolution. Finally 9 antigens with a total of 20 epitopes are obtained.

**Figure 6:**
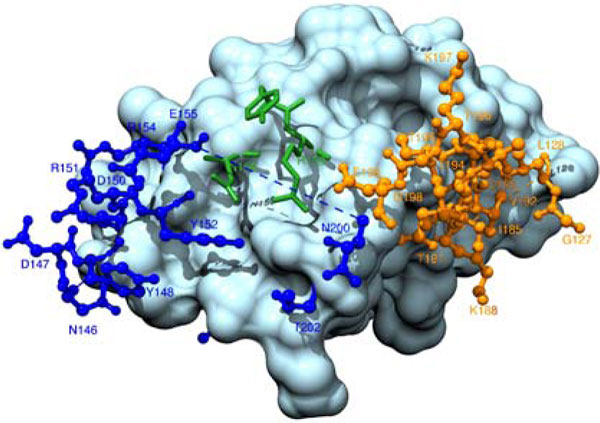
**Two epitopes with 3 overlapping residues on the prion protein**. The 17 epitope residues within PDB entry 1TQB are colored in orange, while the 15 epitope residues in PDB entry 2W9E are rendered in blue. The residues with green color are the three overlapping epitope residues.

BeTop can identify 8 out of the 9 antigens with multiple epitopes; and for all of the 20 epitopes, BeTop can detect 19 of them. However, the conventional approaches would take the union of all the epitope residues on an antigen as a single epitope. The average performance of sensitivity, specificity, f-score and accuracy of applying BeTop to multiple epitope prediction are 0.393, 0.907, 0.321, and 0.858, respectively. As an example, BeTop achieves an averaged f-score as high as of 0.611 in identifying the two epitopes on the prion protein (Figure 6). Detailed performance is available at Additional File 2: Table S2.

As expected, BeTop can identify as many epitopes as possible when they exist on an antigen. For instance, there are four epitopes on the antigen hen egg white lysozyme. BeTop can detect all of the four epitopes with an average f-score and accuracy of 0.376 and 0.849. These experimental results show that multiple epitopes predicted by BeTop are not false positives, and it does not mix up multiple epitopes either.

### Graphical triplet patterns for epitopes

**Figure 7:**
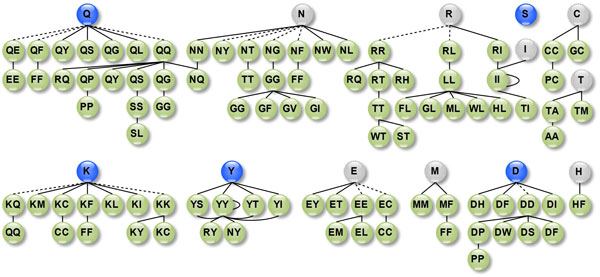
**Features that favor the epitope class**. Here a feature is represented by two nodes connecting with a solid line. Two nodes with patterns XY and YZ form a complete triangle XYZ, while patterns X and XY form only a contacting residue pair XY. The blue-filled nodes are single residues in favor of the epitopes while the gray-filled nodes are not.

**Figure 8:**
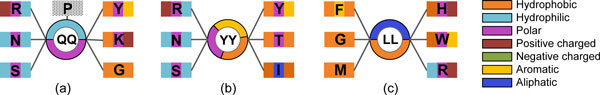
**Examples of triplet feature patterns rich in the epitopes**. The single letters in each colored rectangle is a residue and the double-letter circles stand for contacting residue pairs. Different colors represent distinctive properties of residues. The edges connecting a residue pair and a single residue form interacting residue triangles. For instance, (a) shows a set of residue triangles including QQR, QQN, QQS, QQY, QQK, QQG and QQP that all favor the epitope class.

We are interested in outstanding features that distinguish epitopes from non-epitopes. By transforming epitope and non-epitope subgraphs into feature vectors and selecting distinct features by LIBSVM, we obtained 144 from the 1770 features. See the full details in Additional File 3: Table S3. Features that favor epitopes are shown in Figure 7. Interestingly, residue triangles of the pattern *XXY *(no order constraint), where *X *is a polar residue and *Y *is a hydrophobic or polar residue, are more likely to be epitope residues, but the pattern *XX *itself has no such differentiability. This type *X *of residues include Glutamine (Q), Aspartate (D), Tyrosine (Y) and Leucine (L). For example, residue pair Glutamine-Glutamine (QQ) interacting with residue Arginine (R), Tyrosine (Y), Asparagine (N), Lysine (K), Serine (S), Glycine (G), or Proline (P) are rich in epitopes. But Glutamine-Glutamine itself cannot be used to distinguish epitopes from non-epitopes. Furthermore, these meaningful features indicate some general patterns including polar and hydrophilic homogeneous residue pair surrounded by hydrophobic or polar residues as shown in Figure 8(a), polar and hydrophobic homogeneous residue pair encircled by polar residues as shown in Figure 8(b), and hydrophobic homogenous residue pairs accompanied by hydrophobic residues as shown in Figure 8(c). In contrast, such phenomena are not observed in the features enriched in the non-epitope clusters; see Additional File 4: Figure S1.

To test the statistical significance of these features, we calculated their G-test values [32]. The top ten features that are in favor of the epitopes and the top ten features that are enriched in the non-epitopes in terms of G-test are shown in Table 3. Intriguingly, the top ten features for the epitope class almost all have the form XXY (no order constraint); this observation consolidates the feature patterns we have identified. However, no similar patterns, such as XXY, can be found in non-epitope preferred features; see Table 3 and Additional File 3: Table S3.

**Table 3:** Top ten features that are in favor of the epitope class and also those that are enriched in the non-epitopes in terms of G-test.

epitope		non-epitope	
	
feature	G-test	feature	G-test
SSL	9.12	YF	5.34
GGI	4.92	SAA	4.69
DDW	4.85	KQ	4.19
NGG	4.53	AC	4.04
RRT	4.23	A	3.20
DDF	4.21	EVV	2.74
STT	3.65	EA	2.59
FLL	3.26	FFV	2.54
QQS	3.13	HC	2.35
HF	2.89	N	2.35

## Conclusion

Epitope prediction is an important way to understanding the immune basis of antibody-antigen interactions and is beneficial to prophylactic and therapeutic solutions. In this study, we proposed a novel graph-based model ("BeTop") to predict B-cell epitope by incorporating statistical ideas, graph clustering algorithms and supervised learning approaches. Our experimental results conducted on two data sets of non-redundant antigen-antibody complexes show that BeTop makes great improvements for identifying those planar epitopes and for distinguishing multiple epitopes in an antigen. We have also presented interesting features and triplet feature patterns for the epitopes which will be useful for us to form new hypothesis in the future studies.

## Competing interests

The authors declare that they have no competing interests.

## Authors' contributions

LZ designed the study and drafted the manuscript; LL helped to polish some of the biological ideas; LW, SH and JL supervised the design of the study and revised the manuscript; All authors read and approved the final manuscript.

## Supplementary Material

Additional File 1 **Additional Table S1 -- The performance of BeTop on 92 antibody-antigen PDB complexes**. Click here for file

Additional File 2 **Additional Table S2 -- BeTop performance on multiple epitopes prediction**. Click here for file

Additional File 3 **Additional Table S3 -- 144 features selected to separate epitope clusters from non-epitope clusters**. Click here for file

Additional File 4 **Additional Figure S1 -- Negative features of non-epitope clusters that distinct from epitope clusters**. Click here for file
