# Supplementary material for: B-cell epitope prediction through a graph model
Source: BMC Bioinformatics. 2012 Dec 7;13(Suppl 17):S20. doi: 10.1186/1471-2105-13-S17-S20 (PMC3521413; doi:10.1186/1471-2105-13-S17-S20)
Supplement: Additional File 1 — Additional Table S1 -- The performance of BeTop on 92 antibody-antigen PDB complexes. [file 1471-2105-13-S17-S20-S1.pdf]

Table S1 The performance of BeTop on 92 antibody-antigen PDB complexes.

PDB*	sen	spe	F1	acc	PDB	sen	spe	F1	acc	PDB	sen	spe	F1	acc
1AR1-CD-B	0.846	0.996	0.880	0.988	1TQB-BC-A	0.833	0.833	0.638	0.833	2XQY-GL-A	0.824	0.966	0.609	0.961
1BGX-HL-T	0.480	0.995	0.615	0.964	1V7M-HL-V	0.118	0.945	0.154	0.848	2XTJ-DB-C	1.000	0.642	0.370	0.676
1CL7-HL-I	0.407	0.818	0.458	0.683	1W72-HL-A	0.667	0.977	0.645	0.960	2XWT-AB-C	0.846	0.880	0.603	0.876
1CZ8-HL-W	1.000	0.850	0.700	0.872	1WEJ-HL-F	1.000	0.777	0.488	0.798	2YC1-AB-C	0.455	0.574	0.256	0.554
1DEE-FE-H	0.917	0.381	0.449	0.500	1YJD-HL-C	0.750	0.736	0.367	0.737	2ZCH-HL-P	0.833	0.973	0.714	0.966
1E6J-HL-P	0.500	0.879	0.286	0.857	1ZTX-HL-E	0.500	0.724	0.311	0.693	3B2U-HL-A	0.789	0.847	0.492	0.841
1EGJ-HL-A	0.727	0.822	0.457	0.812	2ADF-HL-A	0.333	0.864	0.200	0.831	3B9K-HL-B	0.706	0.940	0.686	0.906
1EO8-HL-A	0.231	0.997	0.353	0.966	2AEP-HL-A	0.895	0.959	0.667	0.956	3BN9-DC-B	0.700	0.977	0.718	0.954
1EZV-XY-E	1.000	0.788	0.455	0.805	2B2X-HL-A	0.714	0.862	0.417	0.851	3CVH-HL-A	0.647	0.918	0.449	0.901
1FNS-HL-A	0.700	0.742	0.215	0.740	2BDN-HL-A	0.643	0.722	0.474	0.706	3D85-BA-C	0.667	0.907	0.556	0.880
1FSK-IH-G	0.875	0.888	0.609	0.887	2CMR-HL-A	0.562	0.849	0.346	0.826	3DVG-BA-Y	0.857	0.548	0.444	0.605
1H0D-BA-C	0.529	0.971	0.621	0.910	2DD8-HL-S	0.667	0.874	0.462	0.854	3GI9-HL-C	0.600	0.976	0.529	0.963
1IQD-BA-C	0.733	0.738	0.349	0.737	2FD6-HL-U	0.417	0.911	0.263	0.888	3GRW-HL-A	0.966	0.808	0.615	0.830
1JPS-HL-T	0.500	0.928	0.465	0.885	2H9G-HL-S	0.545	0.792	0.429	0.750	3H42-HL-B	0.619	0.958	0.481	0.943
1JRH-HL-I	0.643	0.765	0.429	0.747	2J4W-HL-D	0.833	0.773	0.741	0.794	3HI6-HL-A	0.667	0.660	0.282	0.661
1LK3-HL-A	1.000	0.587	0.375	0.632	2J5L-CB-A	0.917	0.591	0.687	0.706	3KR3-HL-D	0.882	0.814	0.750	0.833
1MHH-DC-F	0.917	0.540	0.478	0.613	2J88-HL-A	1.000	0.819	0.243	0.824	3KS0-HL-B	0.182	0.975	0.267	0.880
1N8Z-BA-C	0.688	0.989	0.667	0.981	2JEL-HL-P	1.000	0.586	0.508	0.659	3L5X-HL-A	0.400	0.989	0.533	0.931
1NFD-HG-D	0.154	0.978	0.200	0.933	2NY1-DC-A	0.385	0.942	0.286	0.918	3L95-BA-X	0.833	0.689	0.303	0.700
1NL0-HL-G	1.000	0.321	0.345	0.424	2NYY-DC-A	0.526	0.986	0.435	0.979	3LDB-CB-A	0.385	0.931	0.250	0.910
1NMB-HL-N	0.500	0.986	0.563	0.964	2Q8B-HL-A	0.700	0.827	0.346	0.818	3LEV-HL-A	0.111	0.881	0.067	0.842
1NSN-HL-S	0.500	0.667	0.269	0.645	2QQK-HL-A	0.800	0.987	0.640	0.983	3LH2-HL-S	0.545	0.736	0.387	0.703
1OAZ-HL-A	0.357	0.960	0.435	0.887	2QQN-HL-A	0.917	0.803	0.431	0.812	3LHP-HL-S	1.000	0.598	0.406	0.647
1OB1-BA-C	0.833	0.881	0.625	0.875	2R0L-HL-A	0.211	0.995	0.333	0.933	3LIZ-HL-A	0.643	0.927	0.391	0.915
1ORS-BA-C	0.300	0.959	0.333	0.909	2R29-HL-A	0.667	0.988	0.769	0.938	3MJ9-HL-A	0.261	0.981	0.364	0.908
1OSP-HL-O	0.882	0.479	0.195	0.506	2R56-IM-B	0.353	0.739	0.200	0.698	3MXW-HL-A	0.444	0.844	0.340	0.797
1OTS-CD-A	0.556	0.986	0.500	0.977	2UZI-HL-R	0.941	0.651	0.376	0.681	3NGB-HL-G	0.840	0.783	0.362	0.787
1PKQ-BA-E	0.812	0.876	0.619	0.868	2VXQ-HL-A	0.692	0.443	0.273	0.478	3NH7-HL-A	0.850	0.508	0.493	0.588
1QKZ-HL-A	0.917	0.761	0.647	0.793	2VXS-IM-A	0.714	0.582	0.222	0.593	3PNW-HG-I	0.765	0.579	0.565	0.636
1R3J-BA-C	0.308	1.000	0.471	0.913	2VXT-HL-I	0.800	0.745	0.381	0.750	3QWO-HL-P	0.875	0.514	0.583	0.623
1RJL-BA-C	0.417	0.590	0.196	0.568	2XQB-HL-A	0.550	0.871	0.524	0.810					

* PDB ID - antibody heavy chain and antibody light chain - antigen chain; sen is sensitivity, spe is specificity, F1 is f-score, and acc is accuracy.
