# Supplementary material for: B-cell epitope prediction through a graph model
Source: BMC Bioinformatics. 2012 Dec 7;13(Suppl 17):S20. doi: 10.1186/1471-2105-13-S17-S20 (PMC3521413; doi:10.1186/1471-2105-13-S17-S20)
Supplement: Additional File 2 — Additional Table S2 -- BeTop performance on multiple epitopes prediction. [file 1471-2105-13-S17-S20-S2.pdf]

Table S2 BeTop performance on multiple epitopes prediction.

antigen	PDB ID	antigen chain	sensitivity	specificity	f-score	accuracy
Hen egg white lysozyme	1A2Y	C	0.267	0.939	0.308	0.860
	1JHL	A	0.636	0.915	0.500	0.891
	1P2C	C	0.250	0.982	0.364	0.891
	3D9A	C	0.444	0.802	0.333	0.752
Vascular endothelial growth factor	1CZ8	W	0.812	0.795	0.578	0.798
	2FJG	V	0.000	0.953	0.000	0.863
Ubiquitin	3DVG	Y	0.429	0.823	0.387	0.750
	3DVG	X	0.600	0.415	0.222	0.440
Influenza virus neuraminidase	1NCB	N	0.316	0.957	0.293	0.925
	1NMB	N	0.105	0.984	0.148	0.941
Von willebrand factor	1FE8	C	0.053	0.929	0.062	0.840
	2ADF	A	0.200	0.885	0.158	0.831
Outer surface protein A	1FJ1	F	0.471	0.756	0.195	0.737
	1OSP	O	0.400	0.948	0.400	0.904
Integrin alpha-L	3EOA	I	0.214	0.703	0.091	0.665
	3HI6	A	0.409	0.703	0.231	0.667
Prion protein	1TQB	A	0.824	0.859	0.651	0.853
	2W9E	A	0.400	1.000	0.571	0.909
Tissue factor	1JPS	T	0.316	0.890	0.267	0.835
	1UJ3	C	0.722	0.952	0.650	0.932
