# Supplementary material for: B-cell epitope prediction through a graph model
Source: BMC Bioinformatics. 2012 Dec 7;13(Suppl 17):S20. doi: 10.1186/1471-2105-13-S17-S20 (PMC3521413; doi:10.1186/1471-2105-13-S17-S20)
Supplement: Additional File 3 — Additional Table S3 -- 144 features selected to separate epitope clusters from non-epitope clusters. [file 1471-2105-13-S17-S20-S3.pdf]

Table S3 144 features selected to separate epitope clusters from non-epitope clusters.

	single residue	residue pair						residue triangle											
positive	K	RI	KI	DF	QL	YS	GC	RRQ	RTT	KQQ	NYY	DDW	QQY	QSS	ECC	YYT	STT	GGV	FLL
	D	KM	NN	DI	EY	YT	MM	RRH	RLL	KCC	NTT	DDS	QQS	QGG	HLL	YYI	TAA	GGI	III
	Q	KC	NW	QY	ET	YI	MF	RRT	RII	KFF	NGG	DDF	QQG	QFF	PCC	WTT	TII	GLL	
	Y	KF	NL	QS	HF	TA	CC	RQQ	KKY	NNQ	NFF	DPP	QEE	EEM	YYY	WLL	GGG	MFF	
	S	KL	DH	QG	YY	TM	II	RYY	KKC	NQQ	DDP	QQP	QPP	EEL	YYS	SSL	GGF	MLL	
negative	N	RL	DV	HT	YA	SF	VV	RRA	KKP	PPI	TFF	LVV							
	A	KQ	EP	HC	YF	GM		RNN	QVV	YWW	GAA								
	L	KP	EA	PW	WG	GV		RDD	EAA	YAA	AVV								
	V	DQ	EV	PA	WV	AC		RHH	EVV	WWA	MMI								
	I	DM	HW	PM	SM	FI		RWW	HHP	SAA	FFV								
