# Supplementary figures and images for: B-cell epitope prediction through a graph model
Source: BMC Bioinformatics. 2012 Dec 7;13(Suppl 17):S20. doi: 10.1186/1471-2105-13-S17-S20 (PMC3521413; doi:10.1186/1471-2105-13-S17-S20)

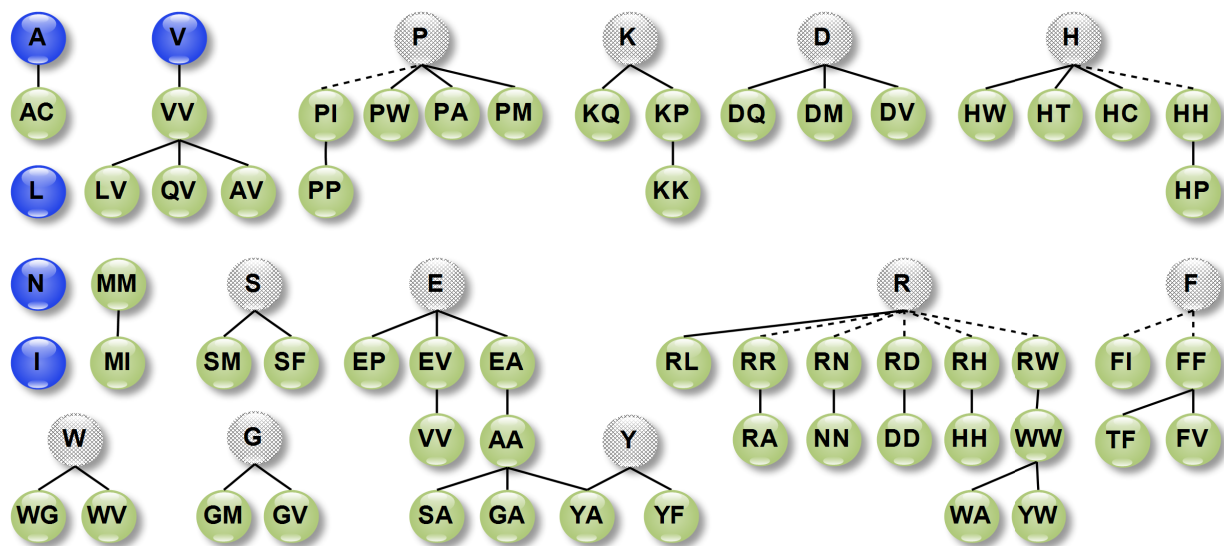

**Figure S1** Negative features of non-epitope clusters that are distinct from epitope clusters.

Supplement: Additional File 4 — Additional Figure S1 -- Negative features of non-epitope clusters that distinct from epitope clusters. [file 1471-2105-13-S17-S20-S4.pdf]
